# Interactions between Inhibitory Interneurons and Excitatory Associational Circuitry in Determining Spatio-Temporal Dynamics of Hippocampal Dentate Granule Cells: A Large-Scale Computational Study

**DOI:** 10.3389/fnsys.2015.00155

**Published:** 2015-11-17

**Authors:** Phillip J. Hendrickson, Gene J. Yu, Dong Song, Theodore W. Berger

**Affiliations:** Department of Biomedical Engineering, University of Southern CaliforniaLos Angeles, CA, USA

**Keywords:** dentate gyrus, compartmental model, oscillations, spatio-temporal patterns, inhibition, associational-commissural fibers, interneurons, topography

## Abstract

This paper reports on findings from a million-cell granule cell model of the rat dentate gyrus that was used to explore the contributions of local interneuronal and associational circuits to network-level activity. The model contains experimentally derived morphological parameters for granule cells, which each contain approximately 200 compartments, and biophysical parameters for granule cells, basket cells, and mossy cells that were based both on electrophysiological data and previously published models. Synaptic input to cells in the model consisted of glutamatergic AMPA-like EPSPs and GABAergic-like IPSPs from excitatory and inhibitory neurons, respectively. The main source of input to the model was from layer II entorhinal cortical neurons. Network connectivity was constrained by the topography of the system, and was derived from axonal transport studies, which provided details about the spatial spread of axonal terminal fields, as well as how subregions of the medial and lateral entorhinal cortices project to subregions of the dentate gyrus. Results of this study show that strong feedback inhibition from the basket cell population can cause high-frequency rhythmicity in granule cells, while the strength of feedforward inhibition serves to scale the total amount of granule cell activity. Results furthermore show that the topography of local interneuronal circuits can have just as strong an impact on the development of spatio-temporal clusters in the granule cell population as the perforant path topography does, both sharpening existing clusters and introducing new ones with a greater spatial extent. Finally, results show that the interactions between the inhibitory and associational loops can cause high frequency oscillations that are modulated by a low-frequency oscillatory signal. These results serve to further illustrate the importance of topographical constraints on a global signal processing feature of a neural network, while also illustrating how rich spatio-temporal and oscillatory dynamics can evolve from a relatively small number of interacting local circuits.

## Introduction

We recently reported on the first generation of a large-scale, biologically realistic compartmental neuron model of the rat hippocampal dentate gyrus (Hendrickson et al., 2015). This model included one million granule cells, axons from 112 k entorhinal cortical neurons (layer II), and 6 k inhibitory interneurons configured synaptically as basket cells of the dentate gyrus. Entorhinal axons provide glutamatergic, excitatory input to granule cells. In the model, basket cells were in appropriate numbers relative to granule cells (i.e., matching an experimentally known ratio), and were arranged in both GABAergic feedforward (relative to entorhinal afferents) and feedback (relative to granule cell efferents) synaptic relations (Seress and Pokorný, 1981; Lübbers and Frotscher, 1987; Zipp et al., 1989). Basket cell axons terminate around the somata and axon initial segments of granule cells, and thus are a powerful source of inhibitory modulation of entorhinal-driven granule cell output (Ribak et al., 1978; Ribak and Seress, 1983).

Dentate afferents from the entorhinal cortex (EC) arise from both its medial and its lateral subdivisions, and terminate in the middle and the outer thirds, respectively, of the dendrites of dentate granule cells (Hjorth-Simonsen, 1972; Hjorth-Simonsen and Jeune, 1972; Steward, 1976; Witter, 2007). The inner third of granule cell dendrites is innervated by associational and commissural fibers arising from neurons intrinsic to the dentate hilus (Berger et al., 1981). Associational fibers arise from neurons ipsilateral to a given hemisphere (Zimmer, 1971); commissural fibers arise from contralateral sites (Gottlieb and Cowan, 1973). Both associational and commissural fibers arise from the same cells of origin within the dentate hilus, namely, mossy cells (though other hilar neurons also may contribute) (Swanson et al., 1981; Ribak et al., 1985). Mossy cells are distributed loosely within the hilus in the rat, but are located more compactly in the polymorphic layer of the rabbit and other species that are higher on the phylogenetic scale, and that have a more well-organized hilar region (Berger et al., 1981; Amaral et al., 2007).

Terminating within the inner one-third of the molecular layer, associational-commissural afferents directly excite granule cells (Buckmaster et al., 1992; Scharfman, 1994; Soriano and Frotscher, 1994; Ribak and Shapiro, 2007). However, inhibitory interneurons embedded beneath the granule cells send their dendrites through the granule cell layer and up into the molecular layer as well (Leranth et al., 1990; Scharfman, 1991). Thus, associational-commissural inputs can directly activate both excitatory and inhibitory components of dentate circuitry (Scharfman, 1995). In fact, it has been shown previously that suprathreshold excitation of dentate granule cells by entorhinal afferents can be determined by the balance between associational-commissural input to excitatory (granule cells) and inhibitory (interneurons) dentate elements (Douglas et al., 1983).

In total, then, at the level of the dentate gyrus there are two major interneuron “loops” contributing to the modification and transmission of entorhinal information to the pyramidal cell populations of Ammon’s horn. The first includes the feedforward and feedback inhibitory pathways enveloping the dentate granule cell population; the second is the mossy cell associational-commissural system. Because mossy cells terminate monosynaptically onto both granule cells and basket cells, there is at least a partial hierarchical organization of the mossy cell system in relation to the basket cell-granule cell feedforward-feedback pathways. The impact of these two control pathways is complex and not easily deduced. A functional understanding of these two interneuron pathways is made even more difficult because selective experimental manipulation of some or most of this circuitry is not readily accomplished with current technology. Transgenic mouse lines and viral vectors can be used to impair the expression of specific synaptic receptors, but they cannot selectively alter specific cell types (Fuchs et al., 2007; Wulff et al., 2009; Korotkova et al., 2010; Murray et al., 2011; Caputi et al., 2012). Optogenetics can be used to modify interneurons to be susceptible to modulation using light, but currently the technique can only target broadly defined and overlapping interneuron groups that are identifiable by the expression of certain molecular markers and would not be able to specifically affect, for example, mossy cells exclusively (Tang et al., 2014; Allen and Monyer, 2015). The nature and relative contributions of the basket cell and mossy cell systems to granule cell output, despite being difficult to elucidate, are remarkably important given that these pathways are the main arbiters of entorhinal input to the CA3/4 pyramidal cell regions. In addition, the dentate hilar zone is the major target for noradrenergic and serotonergic brainstem afferents that have a powerful modulatory influence on hippocampal function (Azmitia and Segal, 1978; Moore et al., 1978; Moore and Bloom, 1979; Loy et al., 1980). These challenges to a more precise definition of basket cell-interneuron and mossy cell-associational pathway functions we feel argues strongly for a mathematical modeling approach. There is a wealth of quantitative anatomical information in the hippocampal literature as to the numbers and densities of neurons and interneurons, morphology of dendrites and distribution of dendritic spines, classes and distributions of synapses, etc., required for such a model to successfully address the issues at hand. Finally, a recent compartmental neuron model of the dentate gyrus, which included the contribution of basket cells and mossy cells to hippocampal epileptic activity, provides guidance for a new, comprehensive modeling study of the functional properties of the associational and basket cell control systems (Santhakumar et al., 2005; Dyhrfjeld-Johnsen et al., 2007).

The goal of the present analysis is to expand our previous large-scale, compartmental neuron model of the dentate gyrus to include both the basket cell-inhibitory circuitry and the mossy cell-associational system. By systematically varying parameters of the mossy cell and basket cell pathways, we hope to provide better insight into their respective and collective functional roles in modifying the dynamics of entorhinal-dentate spatio-temporal activity patterns.

In brief, our analyses revealed that the feedforward component of the inhibitory circuit serves to scale the total amount of granule cell activity, with the ability to shut it down almost completely, while the feedback component has the ability to introduce high-frequency rhythmicity in the network. The associational pathway, in partnership with the inhibitory network, has the ability to both sharpen spatio-temporal clusters that exist due to the connectivity constraints imposed by the perforant path topography, and to introduce new clusters that span a greater portion of the septo-temporal extent of the dentate gyrus. Furthermore, by varying the relative strength of the inhibitory and excitatory associational input to granule cells, it’s possible to introduce high-frequency rhythmicity that’s modulated by a slowly varying oscillatory signal.

## Materials and Methods

### Model Features and Scale

**FIGURE 1:**
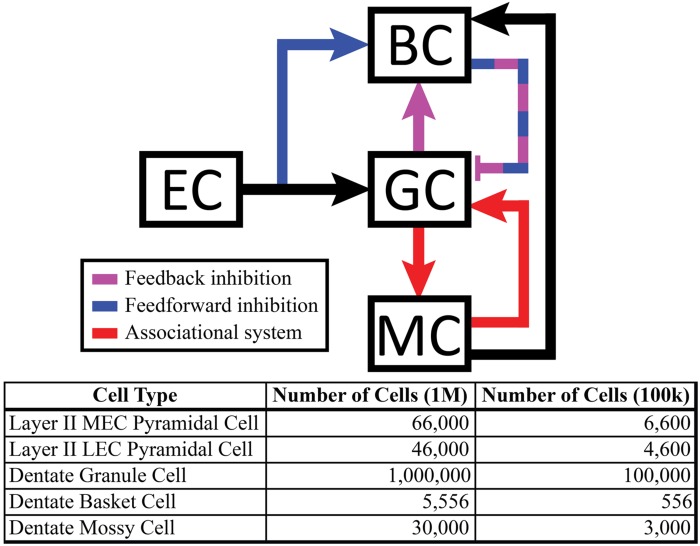
**(Top)** Schematic showing local feedback circuits in the dentate gyrus, with the perforant path providing input to both granule cells and basket cells. The hierarchical nature of local projections in the dentate gyrus can be seen in the mossy cell population, which both excites granule cells monosynaptically and inhibits them disynaptically. **(Bottom)** The number of cells included in the full-scale model matches numbers reported in anatomical studies.

The dentate model consisted of granule cells, basket cells, and mossy cells with input arriving from axons organized as those from layer II neurons of the EC. The EC cell axons provided monosynaptic excitatory input to both granule cells and basket cells. Granule cells provided excitatory input to both basket cells and mossy cells, and basket cells provided inhibitory input to granule cells. Thus, granule cells received both feedforward and feedback inhibition via the basket cell population. Mossy cells provided excitatory input to both granule cells and basket cells (**Figure 1**).

Simulations were performed at two scales: a full scale containing cell numbers equivalent to those found in a single, adult rat hippocampus (one hemisphere) and a reduced scale containing 1/10th of the number of cells. The 1:10 scale was used to decrease computation time. However, all results reported here were observed at both scales. The numbers of cells included in the simulations can be found in **Figure 1**. Simulations were performed using the NEURON (v7.3) simulation environment (Carnevale and Hines, 2006; Migliore et al., 2006); Python (v2.7) was used for model specification, data visualization, and data analyses (Oliphant, 2007; Hines et al., 2009).

### Morphology

Granule cell morphologies were generated using the L-Neuron tool following the methods of Ascoli and Krichmar (2000) and modifications of L-Neuron described in our previous work (Hendrickson et al., 2015). Parameters describing the dendritic arbor of granule cells were obtained from a database of reconstructed granule cells and were input into L-Neuron to create individual, and presumably unique, morphologies for each granule cell modeled. Granule cell models were discretized using NEURON, resulting in individual models that each contained over 200 compartments. Basket cells and mossy cells were modeled as single compartment somata, so they were not conceived of as having any dendritic structures. The decision to include somata only for these two cell types was driven by the relatively low number of dendritic reconstructions available in databases like NeuroMorpho.org from which to draw statistics.

No cells in the model were given explicit axon compartments. Instead, axons were functionally present in the form of propagation delays that postponed the time between the generation of a presynaptic action potential and subsequent initiation of the postsynaptic potential. The delay was calculated using reported action potential conduction velocities and the path length traveled between the pre- and post-synaptic neurons.

### Topography

To generate connectivity between neurons, the densities of axon terminal fields were modeled as distributions that governed the probability of connection to postsynaptic neurons within the target field. The distributions were parameterized to match the anatomical extent of axon terminal fields.

The perforant path projection describing the topographical connectivity between the medial entorhinal cortex (MEC), the lateral entorhinal cortex (LEC), and the dentate gyrus was implemented using data from a study that involved systematic injections of retrograde tracer throughout the septo-temporal axis of the dentate gyrus in rats (Dolorfo and Amaral, 1998). The procedure describing the quantification and implementation of the data to the model’s connectivity is specified in detail in a previous publication (Hendrickson et al., 2015). The MEC and LEC axon terminals were made to synapse within laminar portions of the dentate molecular layer as described by others (Hjorth-Simonsen and Jeune, 1972; Witter, 2007). MEC axons targeted dendritic segments located within the middle third of the molecular layer; LEC axons targeted dendritic segments located within the outer third. The axon terminal field was distributed across the entire transverse extent of the DG and extended 1–1.5 mm in the septo-temporal direction (Tamamaki and Nojyo, 1993).

Granule cells send their axons into the hilus where branching occurs; axon terminals of these branches synapse with cells including, but not limited to, basket cells and mossy cells (Seress and Pokorný, 1981; Lübbers and Frotscher, 1987). The axon terminals of basket cells target the granule cell layer, synapsing with the somata and near the initial axon segments of granule cells (Ribak and Seress, 1983). The connection between basket cells and granule cells comprise the feedback inhibition circuit in our model. The dendrites of basket cells also extend into the molecular layer allowing the EC to innervate the basket cells (Zipp et al., 1989); this provides the feedforward inhibitory circuitry.

**FIGURE 2:**
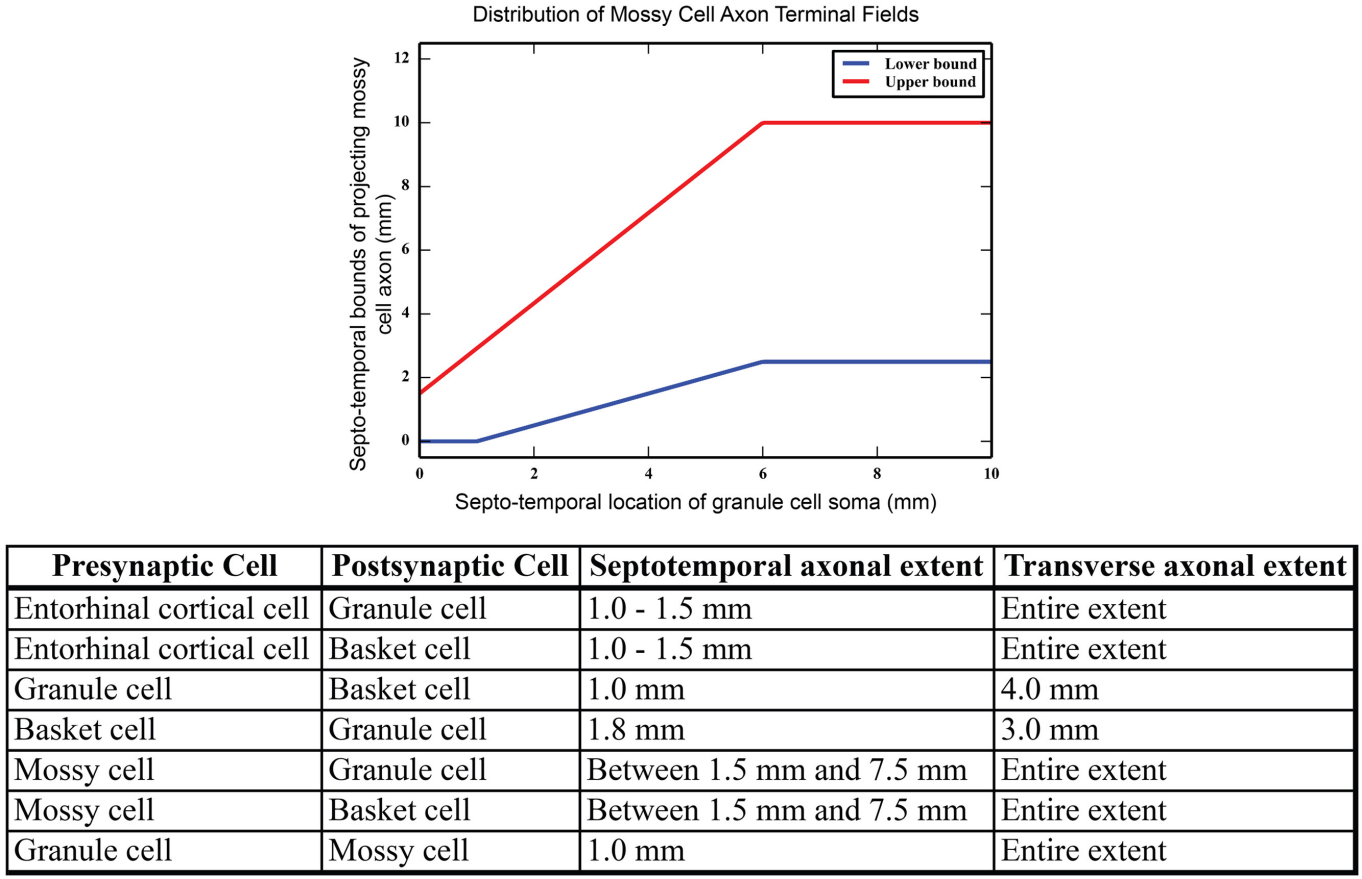
**(Top)** Summary of mossy cell axonal projection, as a function of septo-temporal position in the hippocampus. **(Bottom)** Summary of axon terminal field extents along both the septo-temporal and transverse axes of the dentate gyrus.

Mossy cell dendrites are restricted to the hilus, but their axons extend into the molecular layer, predominantly in the inner third (Buckmaster et al., 1996; Ribak and Shapiro, 2007). The septo-temporal extent of the mossy cell axons changes based on the septo-temporal cell body position. In general, mossy cells located more septally have axons that extend up to 2/3 of the dentate, while mossy cells located more temporally target only approximately 1/3 or less of the dentate gyrus (Zimmer, 1971). The variation of the mossy cell axon extents are summarized in **Figure 2**. Within the inner third of the dendritic field, axon terminals contact both granule cell and basket cell dendrites (Scharfman, 1995), and compose the associational pathway in the network. The parameters for the EC, granule, basket, and mossy cell axon distributions are summarized in **Figure 2**. Connectivity was further determined using anatomically derived convergence and divergence values reported in the literature. Convergence refers to the number of presynaptic inputs that a neuron will receive from a given cell type; divergence refers to the number of postsynaptic targets that a neuron will contact of a given cell type. These values are summarized in **Table 1**.

**Table 1:** Synaptic parameters, including numbers of synapses, synaptic weights, EPSP/IPSP magnitudes and rise/fall times, and reversal potentials.

Synapse counts:
GC # spines – inner 1/3	1150–1350	GC to BC	500–1400
GC # spines – middle 1/3	1050–1200	MEC to BC	100–200
GC # spines – outer 1/3	1100–1300	LEC to BC	100–200
BC to GC	40–80		
MC to GC	750–850	MC to BC	950–1050
**Synaptic weights:**
MEC to GC	1.17E-5	GC to BC	1.13E-4
LEC to GC	1.50E-5	MEC to BC	4.21E-6
BC to GC	1.09E-5	LEC to BC	4.21E-6
GC to MC	2.00E-5	MC to GC	1.17E-6
MC to BC	2.27E-5		
**EPSP/IPSP rise time (ms):**
MEC to GC	1.05	GC to BC	0.1
LEC to GC	1.05	EC to BC	1.05
BC to GC	1.05		
**EPSP/IPSP fall time (ms):**
MEC to GC	5.75	GC to BC	0.59
LEC to GC	5.75	EC to BC	18
BC to GC	5.75		
**Reversal potentials (mV):**
MEC to GC	0	GC to BC	0
LEC to GC	0	EC to BC	0
BC to GC	-75		

### Bioelectric Properties and Pharmacology

Biophysical parameters for each of the cell types in the model were derived from previously published experimental data and from previously published computational models of the DG (Santhakumar et al., 2005; Dyhrfjeld-Johnsen et al., 2007; Hendrickson et al., 2015). **Table 2** shows both the channel distributions for the cell types, and key passive and active properties assumed for each cell type.

**Table 2:** Passive and active biophysical parameters for dentate cells.

Cell Type	Property/mechanism	Soma	GCL	Inner 1/3	Middle 1/3	Outer 1/3
Granule cell	Soma S.A. (cm2)	4.97E-04				
	Soma volume (cm3)	1.11E-06				
	R.M.P. (mV)	-75.01				
	Rin (M-Ohms)	185.86				
	Membrane time constant (ms)	31				
	Latency to first AP (ms)	100				
	Cm (uF/cm2)	9.8	9.8	15.68	15.68	15.68
	Ra (ohm-cm)	210	210	210	210	210
	Leak (S/cm2)	2.90E-04	2.90E-04	4.57E-04	4.57E-04	4.57E-04
	Sodium (S/cm2)	0.84	0.126	0.091	0.056	–
	Delayed rectifier K (slow)	0.006	0.006	0.006	0.006	0.008
	Delayed rectifier K (fast)	0.036	0.009	0.009	0.00225	0.00225
	A-type K (S/cm2)	0.108	–	–	–	–
	L-type Ca (S/cm2)	0.0025	0.00375	0.00375	0.00025	–
	N-type Ca (S/cm2)	1.47E-03	7.35E-04	7.35E-04	7.35E-04	7.35E-04
	T-type Ca (S/cm2)	0.000074	0.00015	0.0005	0.001	0.002
	Ca-dependent K (SK)	0.001	0.0004	0.0002	–	–
	Ca- and V- dependent K (BK)	1.20E-04	1.20E-04	2.00E-04	4.80E-04	4.80E-04
	Tau for decay of Ca (ms)	10	10	10	10	10
	Steady-state Ca (mol)	5.00E-06	5.00E-06	5.00E-06	5.00E-06	5.00E-06
Mossy cell	Soma S.A. (cm2)	2.51E-03				
	Soma volume (cm3)	2.51E-05				
	R.M.P. (mV)	-64.75				
	Cm (uF/cm2)	0.6				
	Ra (ohm-cm)	100				
	Leak (S/cm2)	1.10E-05				
	Sodium (S/cm2)	0.12				
	Delayed rectifier K (fast)	5.00E-04				
	A-type K (S/cm2)	1.00E-05				
	L-type Ca (S/cm2)	6.00E-04				
	N-type Ca (S/cm2)	5.00E-05				
	Ca-dependent K (SK)	1.60E-03				
	Ca- and V- dependent K (BK)	1.65E-02				
	Tau for decay of Ca (ms)	10				
	Steady-state Ca (mol)	5.00E-06				

Synapses were modeled as mechanisms whose activation would result in a conductance change that followed a time-course approximated by the difference of two exponentials. Excitatory connections were represented as AMPA synapses while inhibitory connections were represented as GABA_A_ receptor synapses. For simplicity, neither NMDA nor GABA_B_ receptors were included in the model, though they will be included in the future. Initially, the rise/fall times, maximum conductances, and reversal potentials were initially adjusted to match experimental recordings of excitatory and inhibitory postsynaptic potentials (EPSPs and IPSPs) in the soma of the relevant cell types. However, these parameters resulted in an overtly synchronous network not consistent with biological recordings. An alternate optimization was performed that used the results of an experimental paradigm designed by Douglas et al. (1983) to balance the excitation and inhibition of the associational mossy cell network (see Results).

### Computational Platform

All simulations were run on a high-performance computer cluster consisting of 394 dual quad-core Intel-based nodes and 74 dual hexa-core Intel-based nodes, for a total of 4,040 processor cores. The system has 8.1 TeraB of distributed RAM, 73.1 TeraB of distributed disk space, and a maximum theoretical performance of 38.82 teraflops. All nodes are connected to a high-speed, low-latency 10G Myrinet networking backbone. These nodes are housed, maintained and monitored in facilities operated by the University of Southern California Center for High-Performance Computing and Communications (USC HPCC Center).

## Results

### Granule Cell Response to Random Entorhinal Input: Firing in Spatio-temporal “Clusters”

**FIGURE 3:**
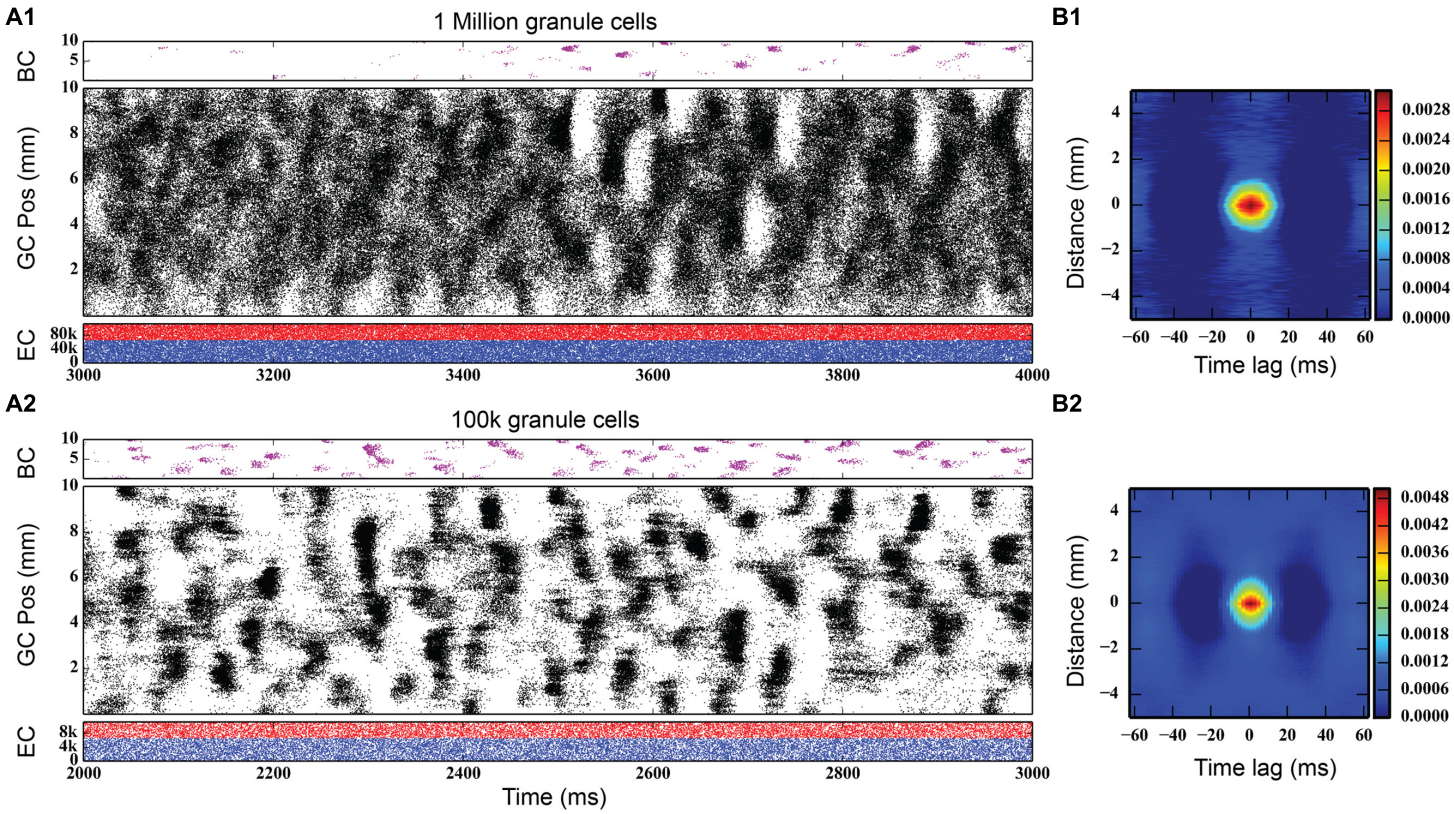
**Simulation results, at two different scales, for topographically constrained EC-DG networks with feedforward and feedback inhibition. (A1)** Simulation results from 1 million granule cells. Despite the random nature of the perforant path input, dentate activity in both granule and basket cells consists of spatio-temporal clusters of spikes. In the million-cell case, only a subset of the full dataset is plotted to keep it from appearing solid black. **(B1)** The spatio-termporal correlation (STC) confirms the existence of clusters. **(A2,B2)** Clusters persist when the network is scaled down to 100 k granule cells.

Initial simulations of granule cell network dynamics to EC input involved both medial and lateral entorhinal (MEC and LEC) neurons firing in a Poisson fashion with a mean frequency of 3.0 Hz, slowly accelerated from 0.0 Hz (by design) over the course of the first 1,000 ms of the simulation. This was performed as a means to reduce what was observed in preliminary studies to be a strong transient response of the system to a step function input if EC input was allowed to start at maximum from zero. The transient response of the network was oscillatory, and the ramping input was a means to mitigate that effect. Four seconds (4.0 s) of neural time were simulated for all conditions presented here. Simulation results with a one million granule cell population revealed that despite continued Poisson EC input, granule cells discharged in a decidedly non-random, non-uniform manner. As shown in **Figure 3** (top), granule cells throughout the entire longitudinal extent of the DG fired in spatio-temporal “clusters,” i.e., local regions of spatio-temporally dense activity. The clusters are organized in irregular patterns persisting for approximately 50–100 ms and separated by periods of lower density activity lasting 50–75 ms. The appearance of clustered spiking in response to Poisson EC input was not specific to million-granule cell populations, but was equally apparent for simulations involving 100 k granule cells as well (**Figure 3**, bottom). Despite the presence of spatio-temporal clusters at both scales, though, there were some differences in network activity. At the million-cell scale, there was more intra-cluster activity in the granule cell population, and sparser basket cell activity, than appeared in the 100 k results.

Quantitative analyses verified the existence of clusters of spike firing. A 2D spatio-temporal correlation (STC) was used, which provided an analysis of the data in both spatial and temporal dimensions. It was constructed by computing every pairwise cross-correlation of discretized spike trains in a random sample of 10,000 neurons. The spike trains were discretized by counting the number of spikes elicited by a particular neuron within a bin size of 5 ms. The resulting cross-correlations were sorted by the distance between the neuron pairs and were further binned using a resolution of 0.05 mm. The mean cross-correlation within each bin was then computed. The STC represents the average spatial and temporal correlation that any particular neuron has with its neighbors.

The right-hand column of **Figure 3** shows the STCs for the million-cell and 100 k cell simulations, respectively. For any given neuron, a spatial correlation persists for 1 mm with a temporal correlation lasting up to 25 ms, resulting in the elliptical STC that is depicted. Furthermore, the spatial and temporal extent of the correlation roughly matches the size and shape of the clusters seen in the raster plots. This analysis verifies the existence of the clusters in the data sets. The similarity in the STC for the million and 100 k cell simulations indicates that the clusters are present at both scales and validates, to an extent, the scaling methodology that was used such that any emergent properties that arise in the million cell simulations would not be lost when scaling the simulations down to 100 k.

### Effect of Feedforward and Feedback Inhibition on Granule Cell Activity

One of the most significant forms of interneuron modulation of granule cell activity has been inhibition due to GABAergic interneurons (Andersen et al., 1964; Kosaka et al., 1984; Gamrani et al., 1986; Sloviter and Nilaver, 1987; Milner and Bacon, 1989; Traub et al., 1996). Given the strong effect that such local interneuron activity is likely to play in modulating granule cell firing, we conducted experiments designed to further examine the role of inhibition on spatio-temporal network activity in the hippocampal dentate gyrus. The inhibition that the basket cell population monosynaptically provides to the granule cells can be initiated through several pathways. In the following sets of simulations, two pathways were investigated: feedback inhibition, due to the innervation of basket cells by granule cells, and feedforward inhibition, due to innervation of basket cells by the perforant path. Several sets of simulations were run to explore the contributions of each of these components of the inhibitory network: one, where the maximum conductance of the basket cell input to granule cells was incrementally increased; two, where the strength of entorhinal input to basket cells was increased; and three, where the strength of the entorhinal input to granule cells was increased. None of the simulations in this section included mossy cells.

At the base level of feedback inhibition with no feedforward inhibition, granule cell activity behaves similarly to previous results. Following a transient response characterized by a slow, large rise and fall of total activity and increased cluster sizes that span up to 8 mm in length, the activity develops into a steady-state consisting of irregular patterns of clusters that are 1–2 mm in size. As the strength of feedback inhibition was increased, however, a marked synchrony in network activity developed, with granule cells across the entire longitudinal extent of the dentate gyrus firing within a 20–30 ms time window followed by a cessation of activity for approximately 30–40 ms. Increasing the inhibition further strengthened this synchrony. These periodic oscillations were caused by a phasic excitation-inhibition cycle maintained by the feedback inhibition between granule cells and basket cells. Basket cells would fire in response to their activation by the granule cells, and this in turn would cause basket cells to inhibit the granule cells to stop firing. Increasing inhibition also decreased the total level of granule cell activity, as reflected in the number of generated spikes over each 4-s simulation; it decreased by 25%, from 742,778 to 557,669.

Discrete Fourier transforms (DFTs) were used to analyze the temporal frequency spectrum of the spike data and quantify the oscillations of the simulations. They were computed using a spike density matrix. The matrix was constructed by counting the total number of spikes elicited within a spatio-temporal bin having a resolution of 0.05 and 8 ms. The DFT was computed for each row of the matrix, resulting in a frequency spectrum for each septo-temporal location on the DG. Because the spectra at each row were similar, the means of all of the DFTs were computed.

**FIGURE 4:**
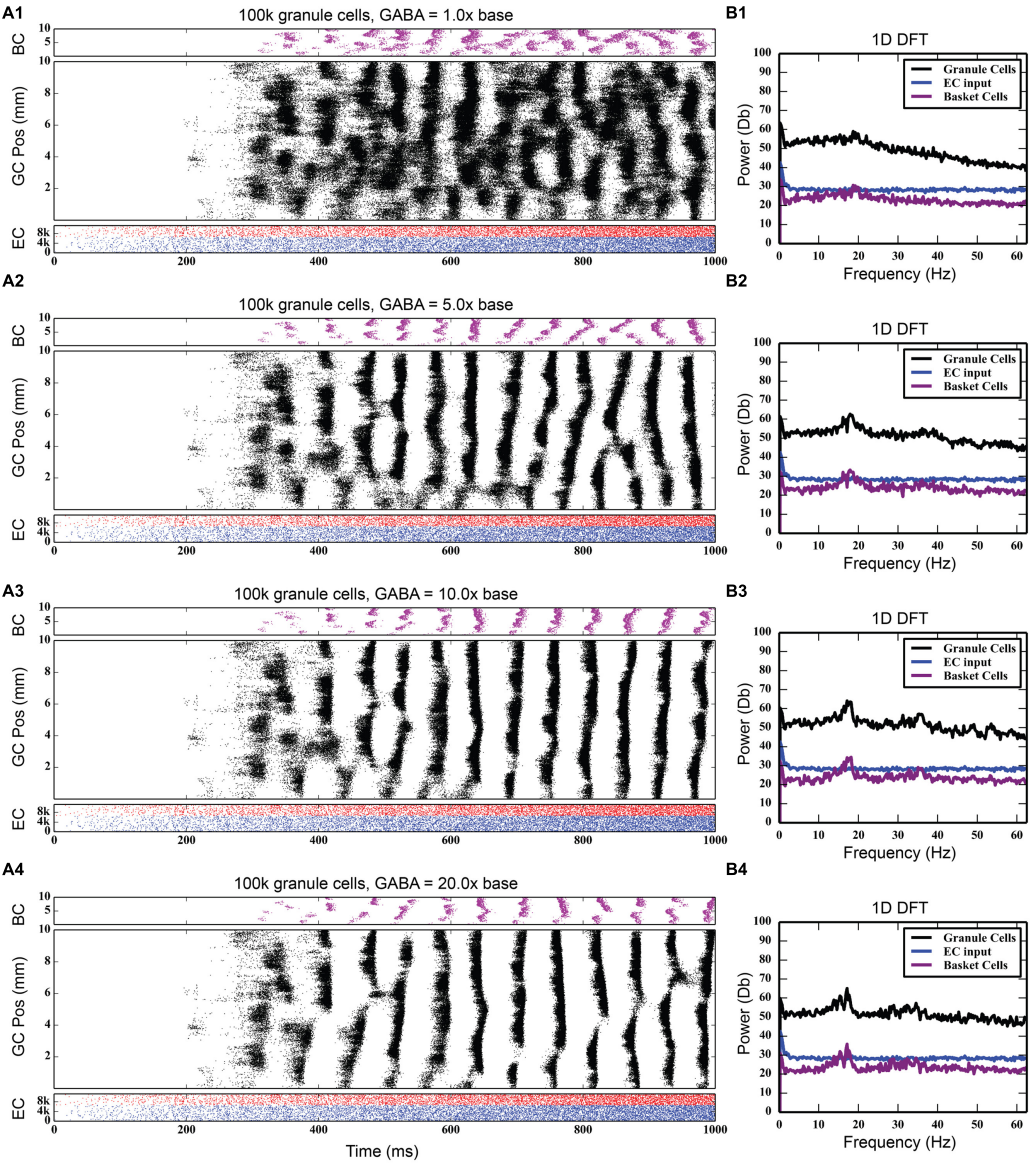
**Effect of progressively increasing strength of feedback inhibition.** As the amount of inhibition increases **(A1–A4)**, pronounced rhythmicity develops in both granule cell and basket cell activity. The presence of this rhythmicity is verified with frequency analysis **(B1–B4)**, which shows a peak developing at approximately 18 Hz.

As **Figure 4** shows, feedback GABAergic inhibition introduced a peak oscillation at 17–18 Hz. Increasing the inhibition did not change the location of the peak but rather sharpened, or decreased the width of the peak. At the highest level of feedback inhibition tested, a smaller secondary peak formed at approximately 35 Hz. The sharpening of the peak and the appearance of the second peak, likely a harmonic of the first peak, are evidence that feedback inhibition introduces oscillation at a certain frequency, but other factors are involved in mediating at which frequency the oscillation occurs, as is demonstrated below.

**FIGURE 5:**
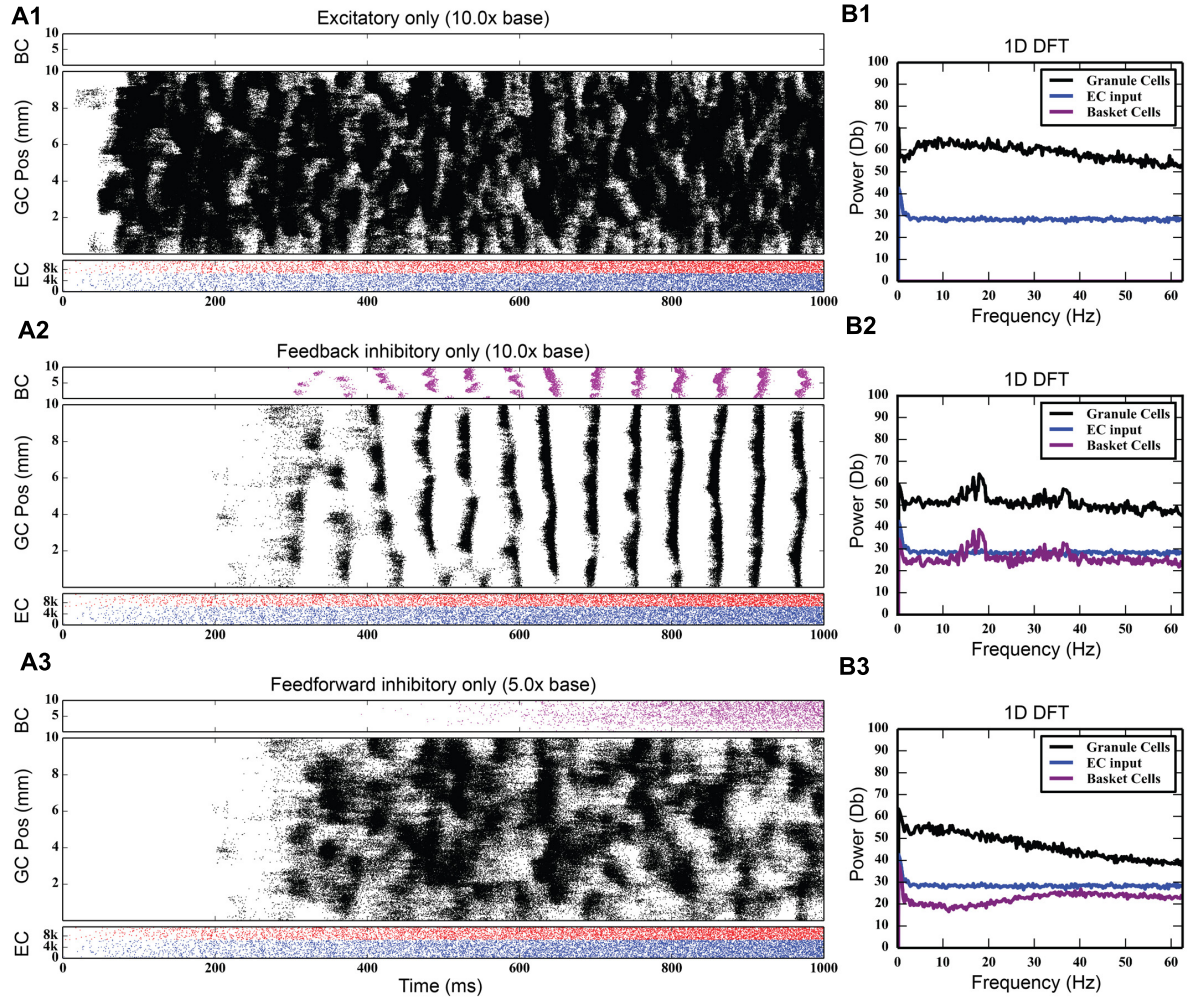
**Large amounts of PP excitation (A1), feedback inhibition (A2), and feedforward inhibition (A3) in isolation, with corresponding frequency plots (column B).** Of note is the fact that only feedback inhibition can cause oscillatory activity in the network.

**FIGURE 6:**
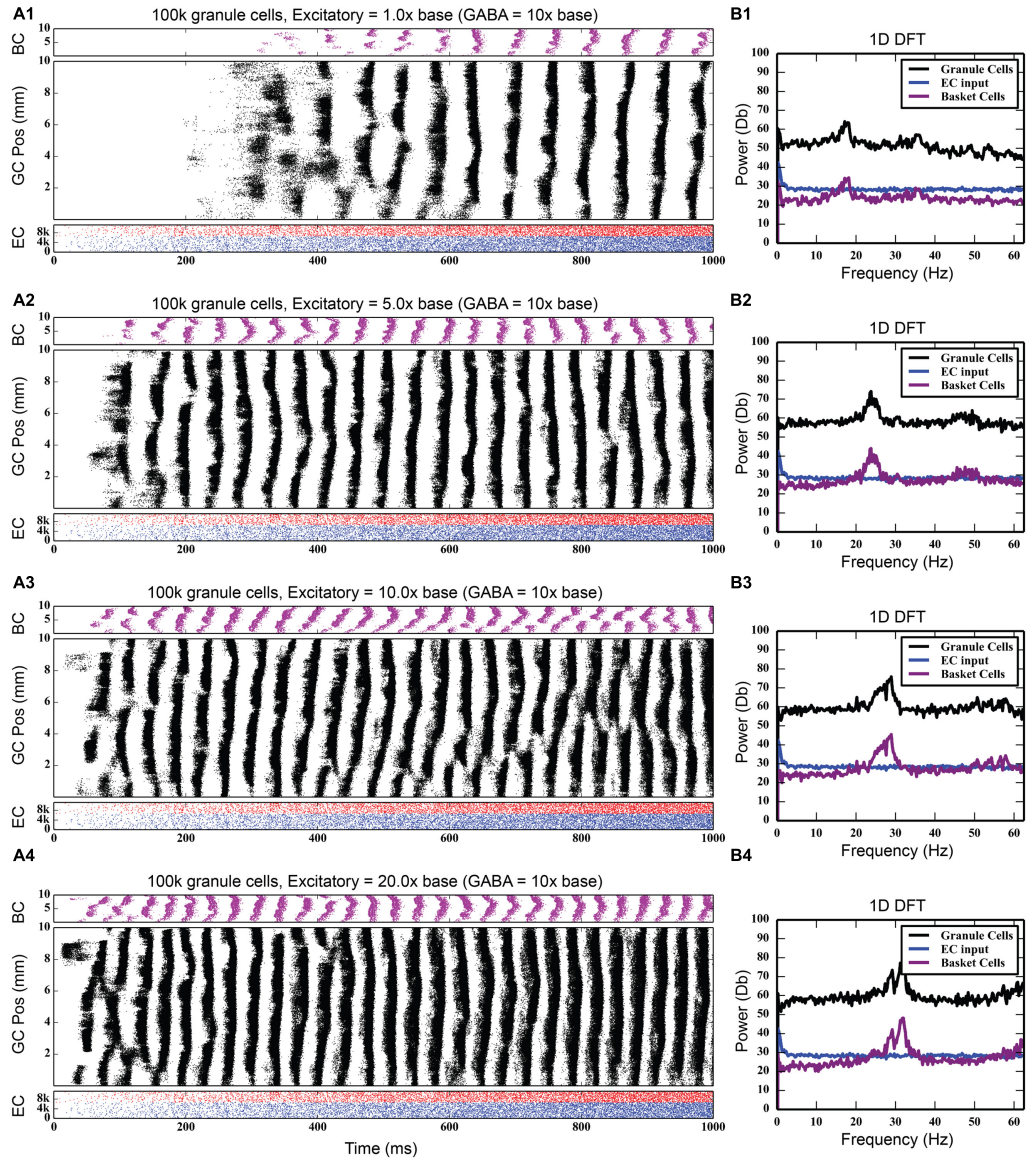
**Effect of increasing strength of perforant path drive in the presence of strong feedback inhibition.** When rhythmicity is present in the network, increasing the strength of the perforant path excitatory drive **(A1–A4)** both strengthens the rhythmicity and increases its freqeuency. As DFT analysis shows, a small 18 Hz peak at the base perforant path strength **(B1)** becomes a much larger peak centered at about 32 Hz when the synaptic weight of the perforant path input is increased by up to 20x **(B2–B4)**.

While the feedback inhibitory circuit appears to be a primary cause of synchrony in the network, the strength of perforant path input to the DG, on its own, did not. Stronger excitatory input only increased the amount of total spiking activity in the granule cell population, increasing it from 742,778 spikes to 4,464,558 spikes over a 10 times increase in PP drive, without ever introducing synchronous activity (see **Figure 5**). However, when synchrony was already present in the network, increasing the strength of the entorhinal input to DG drove the network into both faster and stronger rhythmicity, shifting it from 17 to 18 Hz at its base strength to 31–32 Hz when 20 times stronger (see **Figure 6**). Thus, in the presence of periodic behavior, feedforward excitation was able to modulate the frequency of oscillation using random, uncorrelated activity. However, it was unable to induce oscillatory behavior alone.

**FIGURE 7:**
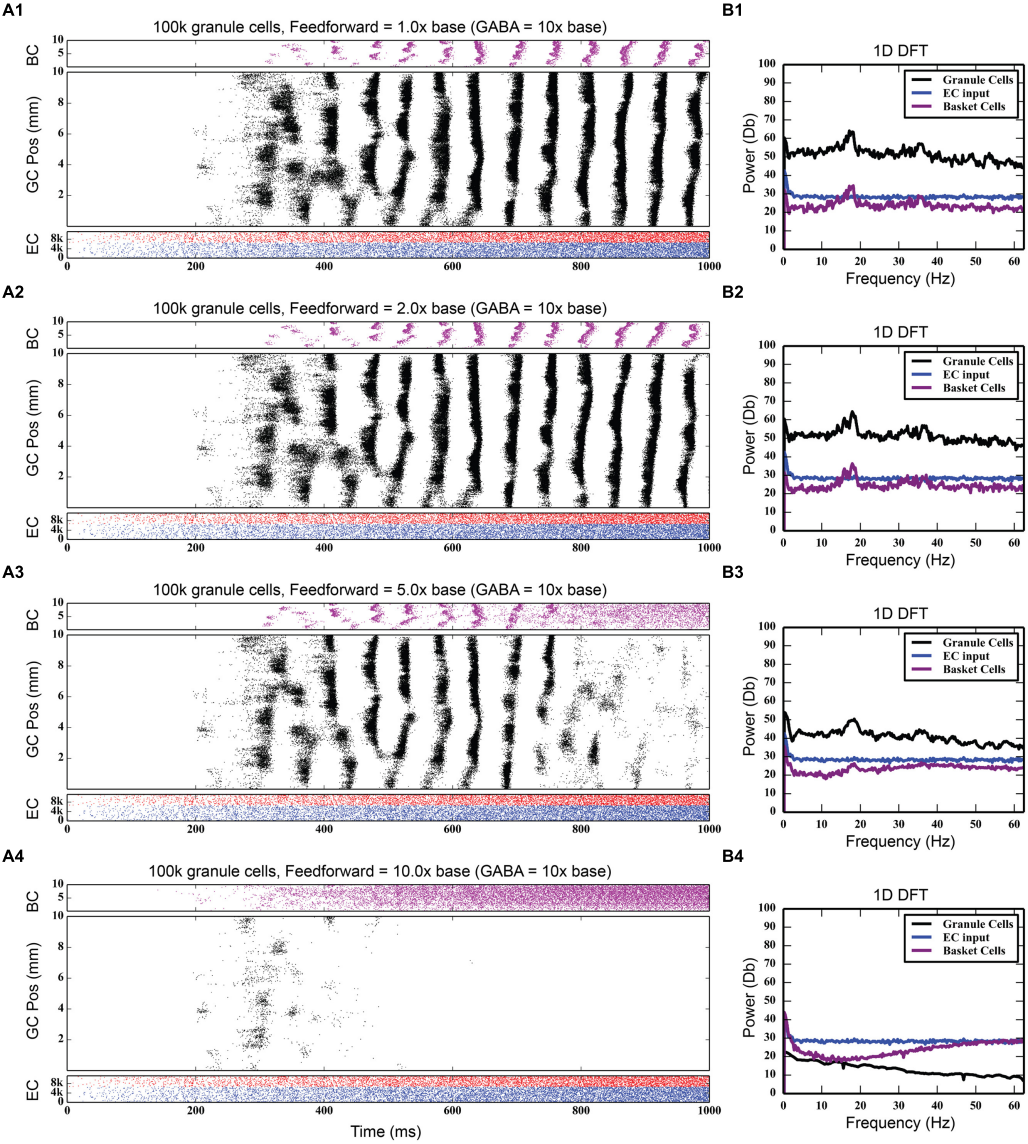
**Effect of increasing the strength of feedforward inhibition.** The feedforward component of basket cell inhibition helps to both desynchronize and scale down total granule cell activity. As the strength of the feedforward inhibition increases **(A1–A4)**, total granule cell activity decreases until there’s almost no activity in the network. The 18 Hz peak in the DFTs **(B1–B4)** also disappears.

Feedforward inhibition had a decidedly different effect on network activity. As **Figure 7** shows, progressively increasing the strength of the perforant path projection to basket cells greatly decreased the overall level of granule cell activity; at its base strength, the granule cell population produced 742,778 spikes over a 4-s period. With five times stronger entorhinal input, only 82,040 spikes were generated, a ninefold decrease in granule cell activity. With 10 times stronger entorhinal input, granule cell activity was almost completely suppressed. The feedforward component of the inhibitory connection was also able to terminate synchrony caused by feedback inhibition as seen in **Figure 7**. Increasing the strength of perforant path input to the basket cells decoupled the basket cells from granule cell activity and prevented the phasic excitation-inhibition that caused the oscillation via feedback inhibition.

### Effect of Associational System Excitation on Granule Cell Activity

**FIGURE 8:**
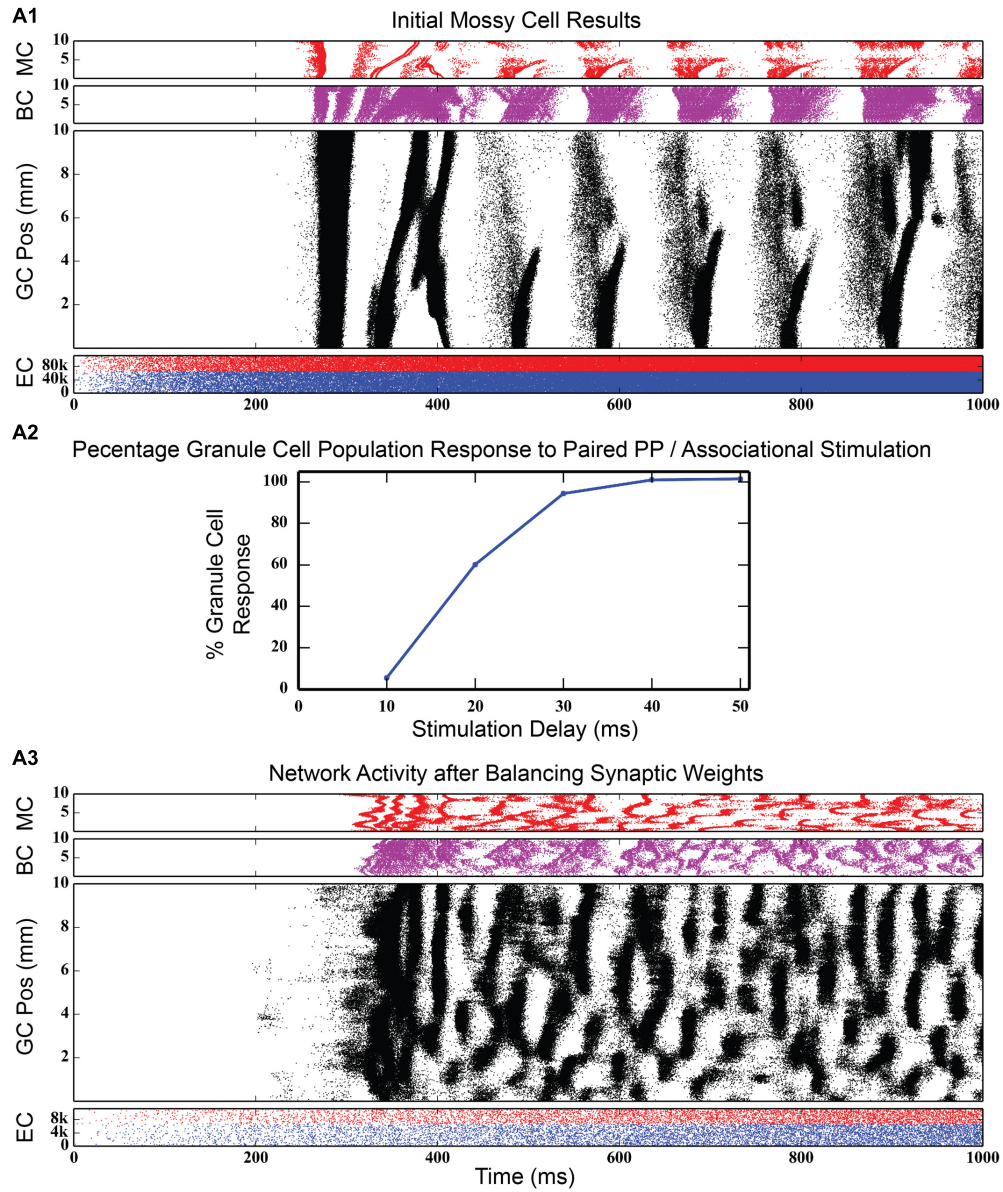
**Initial simulation results after connecting the mossy cell associational pathway.** When EPSP magnitudes are set at values reported in the literature, strong synchrony develops in all three dentate cell populations **(A1)**. When synaptic weights are rebalanced to fit the results of the paired-pulse stimulation experiments of Douglas et al. (1983) **(A2)**, rhythmicity in the network disappears, replaced by a large variety of spatio-temporal clusters **(A3)**.

The dentate gyrus has a known excitatory feedback circuit via the associational pathway. Mossy cells receive input from granule cells and provide excitatory feedback to granule cells. The next series of simulations was designed to study the effect of the interactions between the GABAergic inhibitory and the associational excitatory circuits. However, the associational pathway is more complex than acting merely as a feedback excitation circuit. Mossy cells also innervate basket cells, so that this cell population provides both direct feedback excitation and indirect feedback inhibition to granule cells. Incorporating mossy cells considerably increases the complexity of the model, almost doubling the number of types of synaptic connections and thus, increasing the dimensionality of parameter space to explore. Initial results with the mossy cell network, when EPSP magnitudes between mossy cells and granule cells were set according to values reported in the literature (Scharfman et al., 1990; Scharfman, 1995), was strong synchrony in all three of the cell body populations in the DG. As **Figure 8**, top shows, the granule cell population was the first to fire in response to perforant path stimulation. Those spikes, though low in number, were enough to stimulate a strong burst of activity in the mossy cell population, which, in turn, reinforced the activity in the granule cells. Both populations likely contributed to the burst of activity from basket cells. After a period of approximately 50 ms, the burst of granule cell activity suddenly stopped, either due to basket cell inhibition and/or the synchronized hyperpolarization of the entire granule cell population. This cycle repeated itself every 100 ms, a finding that was corroborated by the frequency analysis, which showed a 10 Hz spike in the GC, BC and MC populations.

#### Re-optimization of Network Parameters

Such a strongly synchronous, highly rhythmic state of dentate firing is non-physiological, according to multiple analyses in freely moving animals (Deadwyler et al., 1976, 1979). In order to move the network to a non-synchronous, less excitatory state, synaptic conductances were rebalanced to correspond with data published from experiments in which both the perforant path and contralateral mossy cell population were stimulated *in vivo* and the granule cell population response was recorded (Douglas et al., 1983). These experimental results show that, while the commissural (and, by extension, associational) afferents to DG have both an excitatory and inhibitory influence on granule cells, the predominant effect is inhibitory: activation of commissural inputs to dentate can prevent perforant path stimulation from reaching threshold. They further show that the amount of inhibition is dependent on the length of the delay between stimulation of the contralateral hippocampus and stimulation of the perforant path. Re-balancing of synaptic weights in the dentate model involved increasing the strength of GABAergic inhibition of granule cells by basket cells while decreasing the strength of the projection from mossy cells to granule cells. To evaluate the re-balancing process, an initial control simulation was run where commissural activation was not simulated, and the total number of granule cell spikes was counted. When input from the commissural pathway was introduced and as the delay between commissural and perforant path input start times was increased, the total number of granule cells spikes was tallied and converted into a percentage relative to the number of spikes generated in the control simulation. The procedure was considered complete when the simulation curve matched that of the experimental findings (see **Figure 8**, middle).

**FIGURE 9:**
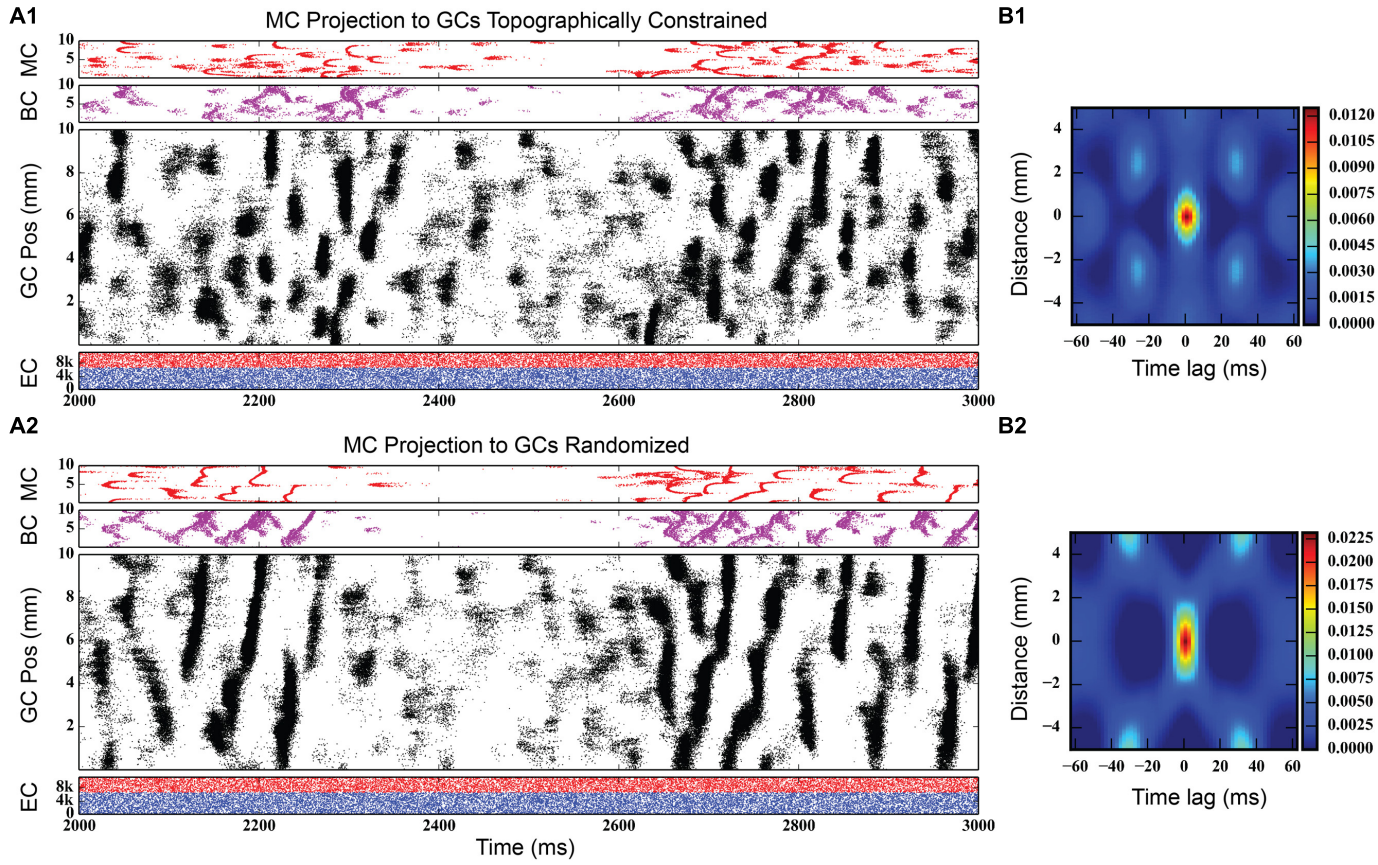
**Simulation results for two dentate networks that only differ in the topography of the mossy cell projection to granule cells. (Top)** Results when mossy cell axonal tree extents vary by their location along the septo-temporal axis of the dentate gyrus, as reported by Zimmer (1971). **(Bottom)** Results when mossy cells are allowed to project randomly to the granule cell population. Note that the size of the spatio-temporal clusters in granule cells increases substantially in the randomly connected network, as evidenced by the spatio-temporal correlations **(B1,B2)**.

**Figure 8**, bottom shows simulation results from the rebalanced network. These results show a pronounced lack of synchrony and, in general, sparser activity throughout the network, though the spatio-temporal clusters present in **Figure 3** (and others) persisted. The granule cell network generated a total of 928,832 spikes over 4 s, a 1.25x increase over the non-associational system network. The clusters, too, have sharper edges (i.e., activity both starts and terminates more suddenly) than clusters from non-associational projection network. The clusters also exhibited a mix of sizes dependent on their septo-temporal location. “Non-associational” clusters tended to remain 1–2 mm in length, but the introduction of associational projections caused larger clusters (3–5 mm) to appear. The larger clusters appeared exclusively in the septal two-thirds of the dentate, which is related to associational projection topography. Associational projections in the septal two-thirds have a greater axon terminal field size (up to 7.5 mm) (Zimmer, 1971), which can introduce spatial correlations spanning a greater distance and result in larger clusters. When local topographic constraints on mossy cell connectivity were removed, the balance of cluster types shifted strongly toward those with a larger spatial extent (see **Figure 9**), a result that emphasizes once again the importance of topography on the development of spatio-temporal cluster functionality (Hendrickson et al., 2015).

In addition, the clusters emerging from mossy cell activity look somewhat different from granule cell clusters driven by only perforant path excitation; instead of an elliptical or ovular shape, mossy cell activity have “C” shaped clusters (see, for example, **Figures 8** and **9**). The “C” shape implies that these clusters have an initiation point where activity begins which spreads almost symmetrically in both directions longitudinally away from this initiation point. The curve of the “C” was due to the temporal delay of the activity as it spread from the initiation point.

**FIGURE 10:**
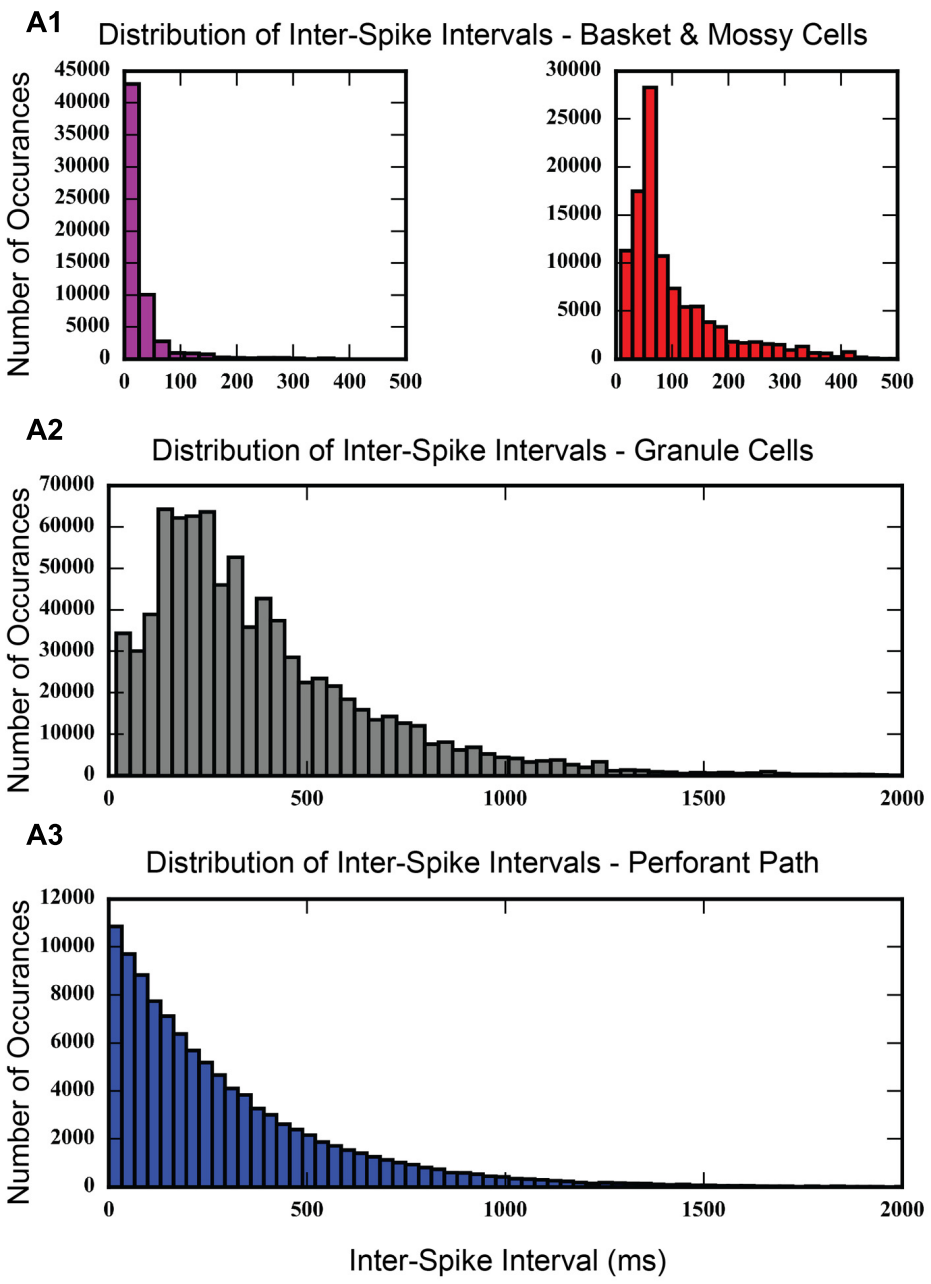
**Histogram showing inter-spike intervals for each of the cell populations in the model.** Inter-spike intervals are very small for basket cells and mossy cells **(A1)**, which makes sense, given that basket cells are fast-spiking interneurons. Granule cells do not exhibit many small inter-spike intervals **(A2)**, but rather, show a peak in the 200–300 ms range. The distribution of inter-spike intervals for entorhinal cortical cells looks exponential **(A3)**, which is expected for a group of cells whose action potentials follow a Poisson process.

An inter-spike interval analysis of the dentate network with associational projections was calculated. As **Figure 10** shows, the histogram of inter-spike intervals for the perforant path input follows an exponential curve with a long tail. Inter-spike intervals range from as small as 0.1 ms to as large as 3,194.9 ms, thus spanning a wide range of frequencies. The granule cell population shows a distribution of inter-spike intervals with a long tail, similar to the perforant path input, but with much fewer small intervals. It has a peak in the 200–400 ms range. Basket and mossy cells have a large concentration of inter-spike intervals less than 100 ms, which drops off almost entirely by 400–500 ms.

At a population level, a unique pattern of activity emerged that was not present in the network without the associational system. More evident in the interneuron activity, there were regions of inactivity lasting approximately 200–250 ms that were interposed between regions of activity. During these silent intervals, the granule cell population also exhibited a reduced activity. However, granule cell clusters still persisted whether or not the population was in its higher or lower activity states, though the clusters were shorter longitudinally during the low activity states. This highlights the influence of mossy cells on cluster size. While mossy cell activity is present, granule cell clusters are larger than when mossy cells are silent. This overall pattern of high/low or active/inactive states represents a slower oscillation that had not been seen in any simulations until the introduction of the mossy cell-based associational system.

#### Evaluation of Selected Pathway Functions

**FIGURE 11:**
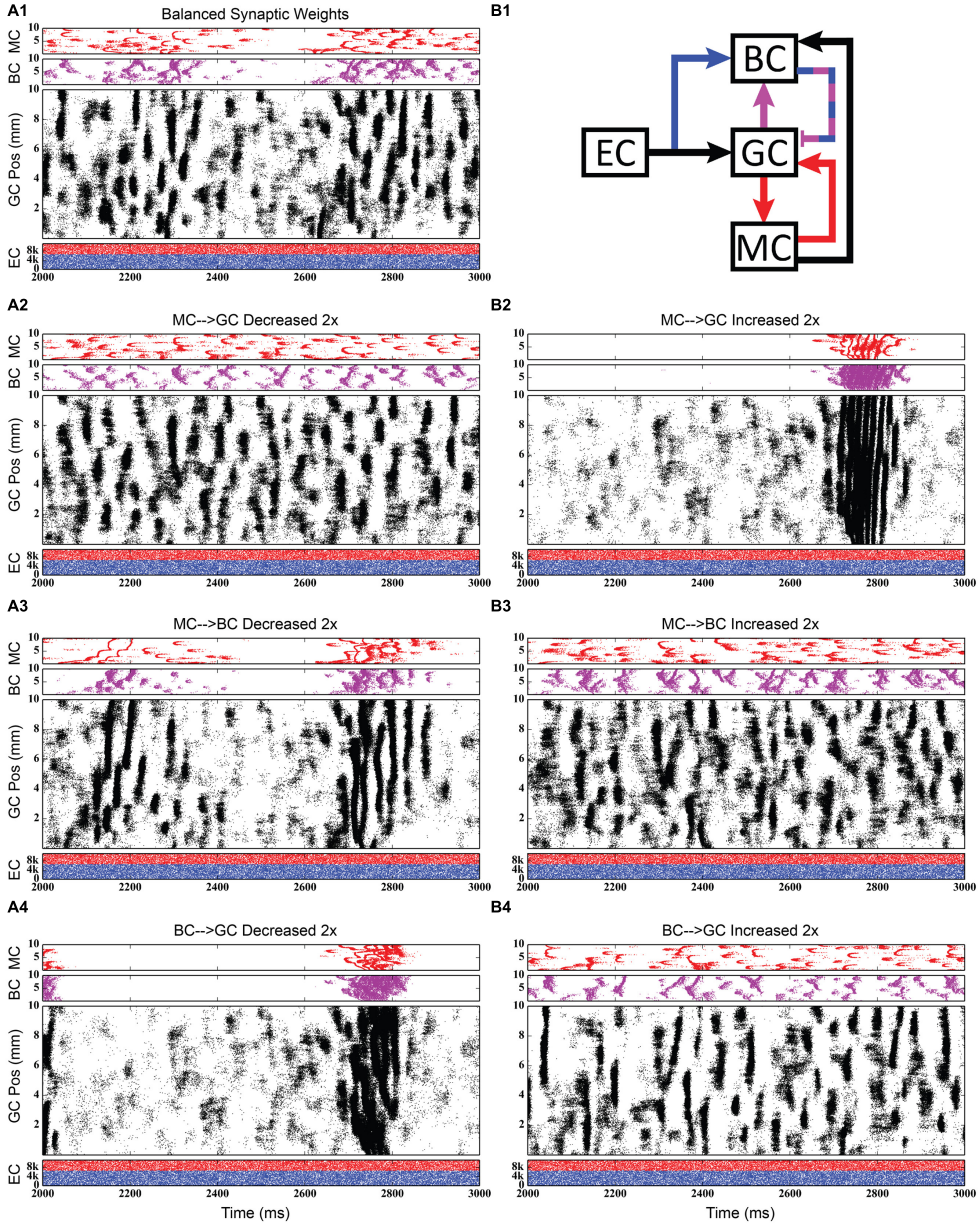
**Simulation results when strengthening and weakening three individual synaptic connections in the granule-mossy-basket cell network.** All raster plots show 1,000 ms of activity. **(A1,B1)** Shows balanced synaptic weights. **(A2,B2)** Show alterations to the MC-GC synaptic weights. **(A3,B3)** Show alterations to the MC-BC synaptic weights. **(A4,B4)** Show alterations to the BC-GC synaptic weights. When the excitatory mossy cell loop is strengthened relative to the inhibitory basket cell loop, a pattern of theta-modulated gamma oscillations develops **(A3,A4,B2)**.

**FIGURE 12:**
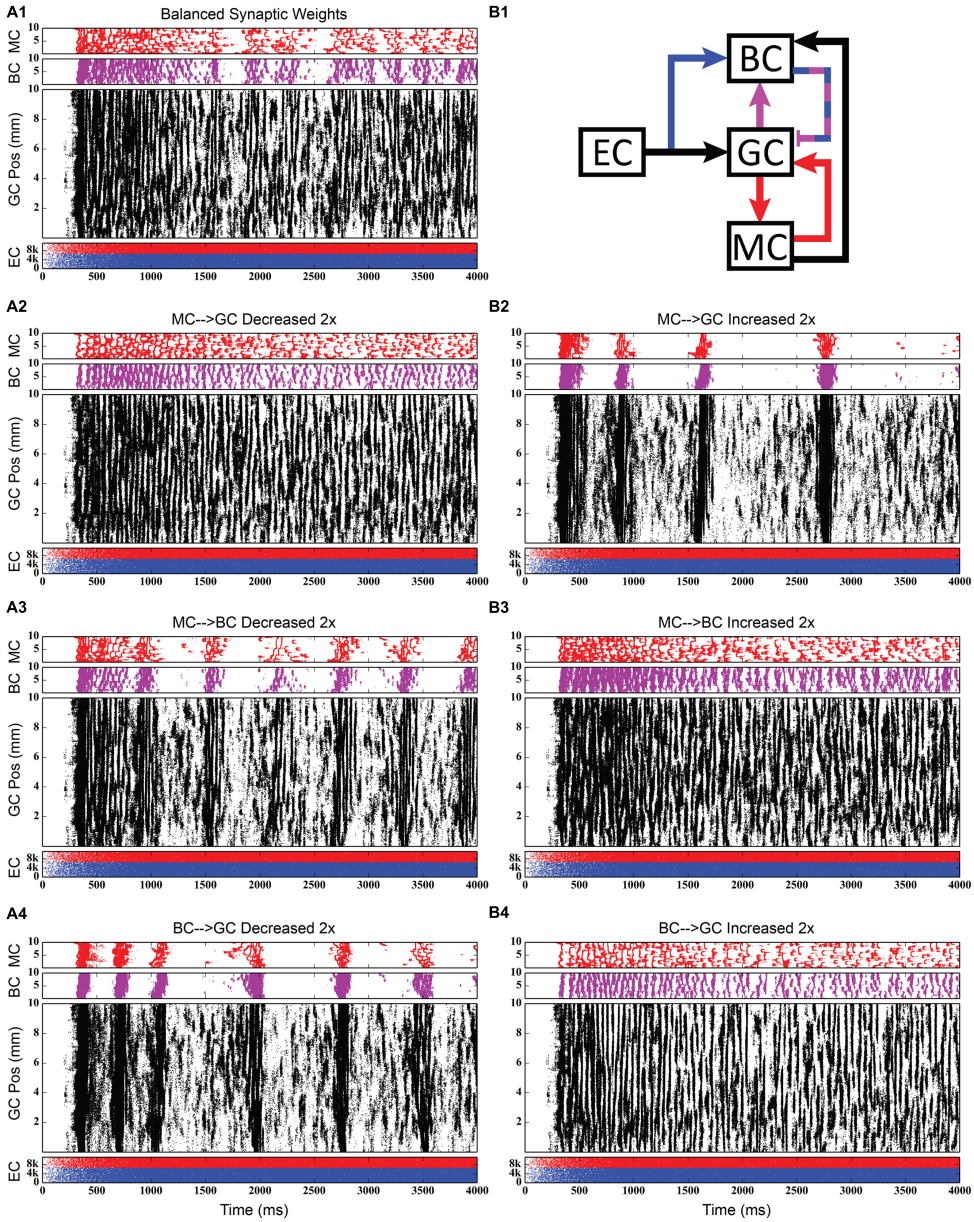
**Same simulation results as in **Figure 11**, but showing 4,000 ms of activity to give a better view of the theta-modulated gamma oscillations. (A1,B1)** Shows balanced synaptic weights. **(A2,B2)** Show alterations to the MC-GC synaptic weights. **(A3,B3)** Show alterations to the MC-BC synaptic weights. **(A4,B4)** Show alterations to the BC-GC synaptic weights.

**FIGURE 13:**
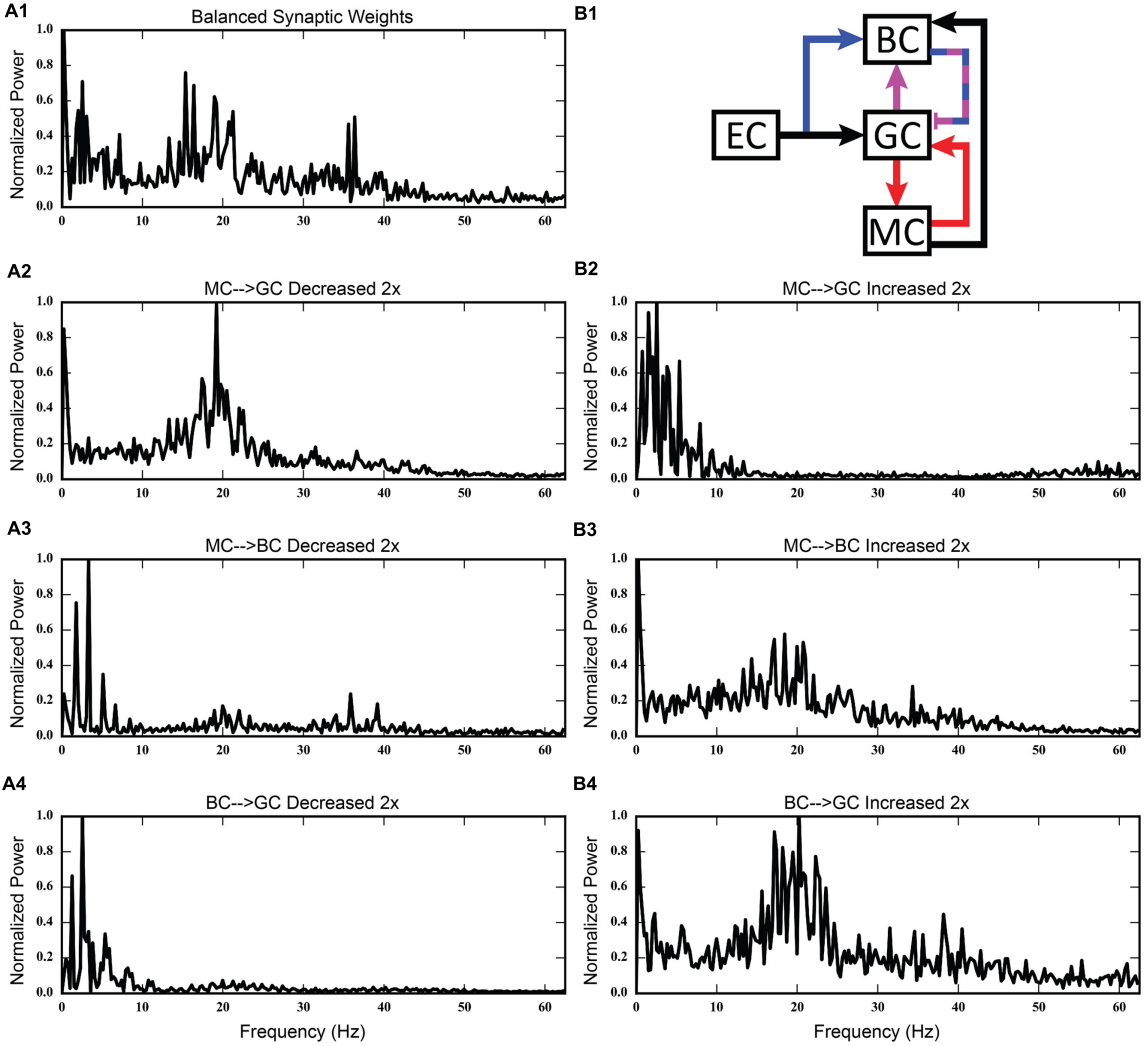
**Discrete Fourier transforms (DFTs) for the data shown in **Figures 11** and **12**. (A1,B1)** Shows balanced synaptic weights. **(A2,B2)** Show alterations to the MC-GC synaptic weights. **(A3,B3)** Show alterations to the MC-BC synaptic weights. **(A4,B4)** Show alterations to the BC-GC synaptic weights.

Using the rebalanced synaptic conductances, a series of experiments were performed to observe the effects of altering the strengths of individual connections between cell types. The rebalanced synaptic conductances will be referred to as the “base weights” when comparing the next set of simulations. While all seven synaptic connections were manipulated, only three of those manipulations are reported on in detail here. See **Figures 11–13** for those details – **Figure 11** shows a 1,000-ms view of each of the relevant datasets, **Figure 12** shows a 4,000-ms view of those same data, and **Figure 13** shows the DFTs for each of the datasets. See **Table 3** for a summary of the effects of manipulating each of the seven connections.

**Table 3:** Summary of changes in network activity due to strengthening and weakening individual synaptic connections.

Connection	Increase/decrease weight?	Effect
MC → GC	Increase	Strong high-frequency activity modulated by low-frequency oscillation. High-frequency bursts are highly synchronous.
	Decrease	Less, but more regular GC activity; spatio-temporal clusters slightly more “fuzzy;” fewer long (3–4 mm) clusters.
GC → MC	Increase	Slight increase in both mossy and basket cell activity, with corresponding decrease in granule cell activity. Otherwise, little discernable difference in activity.
	Decrease	Slight decrease in both mossy and basket cell activity. Otherwise, little discernable difference.
MC → BC	Increase	GC activity is more regular; no other strong differences.
	Decrease	Strong high-frequency activity modulated by low-frequency oscillation. Low frequency component is higher than in MC → GC and BC → GC cases, while high frequency component is slower.
GC → BC	Increase	Very regular activity in all three dentate cell populations.
	Decrease	Low-frequency oscillation modulates strength and density of spatio-temporal clusters in granule cells.
BC → GC	Increase	Spatio-temporal clusters have sharpter tailing edges, with less inter-cluster activity.
	Decrease	Strong high-frequency activity modulated by low-frequency oscillation. Synchrony in high-frequency activity not as strong as for MC → GC case.

The second row of **Figures 11–13** shows the result of both strengthening and weakening the direct, excitatory projection from mossy cells to granule cells. When the mossy cell drive to granule cells is decreased, regions of interneuron inactivity no longer appeared. Likewise, there were no periods during which the granule cell population underwent a lower activity state. Otherwise, the granule cells still exhibited clustered behavior, and the “C” clusters in the mossy cells remained. This was similar to the network activity in the previous network during regions of interneuron activity. Total granule cell activity also decreased by 22%. Increasing the strength of the excitatory feedback to granule cells extended the length of interneuron inactivity significantly. Though the length of the silent interval was similar to that of the base weights near the beginning of the simulation, the interval increased in length from 250 to 1,200 ms by the end of the simulation. During the silent intervals, granule cells exhibited sparse but clustered activity patterns, similar to that found for the base weights. However, the patterns generated by all cell types during intervals when the interneurons were active changed dramatically. These active interneuron intervals, lasting 100–200 ms, are composed of multiple waves of synchronous activity. Although most of the activity is concentrated during the active interneuron intervals, the total granule cell activity increased by 27%, indicating that the activity during the active interneuron intervals is dense.

The third row of **Figures 11–13** shows the result of manipulating the strength of the mossy cell projection to basket cells. This change affected the inhibitory aspect of the associational pathway in the network. Halving the strength of the mossy-basket connection enhanced the silent interneuron intervals relative to the network with base synaptic weights. The intervals increased in length to 250–350 ms but also became more numerous; the active interneuron intervals became shorter. Also, granule cell activity during the silent interneuron intervals remained clustered. The direction of this change is similar to that when the MC-GC connection was strengthened, though there were differences. Instead of exhibiting bands of activity that extended across the entire longitudinal extent, granule cell clusters of many sizes occurred, ranging from 1 to 8 mm. Furthermore, the intervals had a greater regularity than the MC-GC case which had silent intervals that increased in length over time. Total granule cell activity increased by 12%. In both instances (i.e., either weakening the MC-BC connection or strengthening the MC-GC connection), the net effect of these changes was to tip the balance toward the associational excitatory loop relative to the inhibitory loop. However, changing the MC-GC connection affected a direct excitatory connection while changing the MC-BC connection altered an indirect inhibition as well as causing disinhibition. Doubling the strength of the mossy-basket connection resulted in a similar effect as decreasing the strength of the mossy-granule connection. The silent interneuron intervals disappeared, and the clustering behavior remained unchanged. Granule cell activity decreased by 14%.

The last row of **Figures 11–13** shows the result of manipulating the strength of the basket cell projection to granule cells. This affects both the basket-granule feedback circuit as well as associational inhibition as the basket cells mediate inhibition for both circuits. Decreasing the strength of basket-granule inhibition had an effect similar to strengthening mossy-granule cell connection; short periods of high activity occurred, interspaced with longer periods of little to no activity. Again, weakening BC inhibition caused the network to favor the associational excitatory loop over of the inhibitory loop; decreasing the BC-GC inhibition disinhibited the network. However, the silent interneuron interval was much greater when the BC-GC inhibition was decreased. Initially having a length of 600 ms, the next silent interneuron interval lasted for 2,000 ms. Furthermore, total granule cell activity increased by 49%, which was greater than the increase caused by the MC-GC manipulation. Another difference lay within the patterns of activity during the active interneuron interval. Granule cell activity occurred as unconnected but distinct clusters as opposed to unbroken bands that spanned the entire longitudinal extent. Following the above trend, increasing the strength of the basket cell projection to granule cells had a similar effect as decreasing the mossy cell projection to granule cells. The silent interneuron interval disappeared and the total granule cell activity decreased by 18%. Because the basket cells were driven by both granule cells and mossy cells, the sensitivity of the BC-GC connection may be greater than the MC-GC connection, which was driven by granule cells alone, and this could account for the more drastic changes that occurred by varying the BC-GC connection.

## Discussion

The dentate gyrus is an integral part of the hippocampal formation, as it both transforms incoming information from the EC and transmits it to subsequent areas of hippocampus. This circuit plays a critical role in the formation of new long-term memories and has two local feedback loops that modulate its activity. Our goal with this study has been to explore the effects of both the inhibitory and associational circuits on network level activity in the dentate gyrus. With respect to inhibitory feedback, basket cells were chosen for the initial implementation of the model as it is the most numerous and most well-known inhibitory feedback element. With respect to the associational system, mossy cells are regarded as the main source of these projections, though evidence exists that other interneurons also contribute to this pathway (Amaral, 1979; Berger et al., 1981; Swanson et al., 1981). These loops are organized in a hierarchical fashion, which makes it difficult to determine experimentally the role that each plays in the spatio-temporal spiking dynamics of granule cells. A mathematical model, however, lends itself exactly to this kind of study because cellular properties and synaptic connections can be selectively manipulated to observe their effect on network function.

### Effects of Inhibitory Interneuronal Circuitry (Feedforward and Feedback)

Our computational model has revealed two key roles for the inhibitory interneuronal circuitry. First, feedback inhibition is necessary to generate rhythmicity in the granule cell population. In generating that rhythmic activity, the feedback inhibitory loop fundamentally changes network activity; instead of generating spatio-temporal clusters spanning 1–2 mm of the septo-temporal extent of the hippocampus, activity now occurs as bands that span its entire length. Similar large-scale spatial synchronization of principle cells, due to GABAergic feedback inhibition, has been shown in network models of CA3 (Traub et al., 1987) and of visual cortex (Bush and Douglas, 1991). In both of those models, however, the presence or absence of rhythmicity was studied with cells that exhibit strong bursting activity. In the present model, rhythmicity develops despite the lack of bursting spike behavior in principal cells. Evidence of GABAergic interneuron–interneuron synapses (Cobb et al., 1997) inspired additional studies that investigated inhibitory interneuron coupling on gamma generation which demonstrated an important role for these synapses in a more robust generation of gamma over a broader range (Bartos et al., 2001, 2002). Basket cell-to-basket cell coupling was not incorporated in the present study.

Other studies have focused on interconnected networks of interneurons as the source of large-scale synchrony in neuronal networks (Traub et al., 1996; Wang and Buzsáki, 1996). Those computational studies found that, among the factors that influence the frequency of rhythmicity, strength of afferent input plays a role. The results of this current study corroborate these prior findings – in the presence of strong feedback inhibition, perforant path excitation of granule cells both strengthens and increases the frequency of rhythmicity. However, unlike those models, we find that, when paired with Poisson distributed spiking input from the EC, feedback inhibition from basket cells, on its own, is also sufficient to generate rhythmicity – no additional interneuron types are needed.

The second key finding from our study of inhibitory interneuron circuitry is that feedforward inhibition plays an important role in scaling the total amount of granule cell activity. At progressively higher levels of feedforward inhibition, granule cell activity decreases until it is almost completely inhibited. This phenomenon can be explained by the fact that entorhinal input to the basket cell population helps to decouple it from the granule cell activity that normally drives it. The scaling effect of feedforward inhibition has been shown in a simple circuit previously (Buzsáki, 1984). In that review paper, the author hypothesized that feedforward inhibitory mechanisms, where they occur in the hippocampus, can be important from an external control standpoint – they can allow other areas of the brain to effectively shut off portions of the hippocampus. This is especially true in the dentate gyrus, which is the first area of the hippocampus that signals originating in other portions of the brain see. Based on these results, feedforward inhibition could be hypothesized to act as a homeostatic mechanism to prevent overt synchronized oscillation and hyperexcitability in granule cells. Increased activation of granule cells would also increase GABAergic inhibition. Also, in the presence of synchronized oscillation, feedforward inhibition can terminate synchrony, as was demonstrated.

### Effects of Associational Circuitry

Adding local associational circuitry to the otherwise large-scale model of the dentate gyrus revealed several key roles for the mossy cell population in shaping the spatio-temporal dynamics of granule cells. First, as was clear in our previous study of entorhinal projections to the dentate, topography of associational connections was found to play an important a role in shaping the clusters of spiking activity in the granule cell population (Hendrickson et al., 2015). In addition to the 1–2 mm clusters that appear from the topographic organization of the perforant path projection, new clusters that span 4–6 mm of the septo-temporal extent of the hippocampus appear when associational connections are included. These clusters form predominately at the septal end of the hippocampus, where mossy cell axonal fields have a similarly large spread. When projections from mossy cells to granule cells are randomized, small clusters virtually disappear anywhere there is mossy cell activity, and are replaced with clusters that have a very large spatial extent.

Second, clusters of granule cell activity evoked by perforant path input had boundaries that were considerably “sharper” in the presence of mossy cell circuitry. This effect appeared to be due to mossy cells evoking a strong response from the basket cell population, which more rapidly inhibited granule cell activity on the trailing end of a given cluster. In addition, because of the relative ease with which granule cells depolarize any given mossy cell to threshold, they facilitate a more sudden onset of spiking activity in the granule cell population at the beginning of each cluster.

Third, incorporating the associational system into the network leads to the appearance of power in the lower theta frequency band of the Fourier transform of the population response. The frequency spectra for the cases in which feedforward and feedback inhibition and perforant path excitatory drive were investigated did not include this lower theta activity. Instead, feedback inhibition gives rise to oscillations in the gamma range, as is consistent with several other computational studies that identified GABAergic inhibition from basket cells as the source of gamma oscillations using single compartment models (Wang and Buzsáki, 1996; Tiesinga and José, 2000; Maex and De Schutter, 2003; Bartos et al., 2007). Only after mossy cells are included in the network does power in the theta range appear. In fact, the simulations demonstrate a theta–gamma network oscillation generated by the interactions of the mossy, basket, and granule cells (**Figure 12**). Through granule cell excitation, the mossy cells mediate both a positive feedback loop, via a direct monosynaptic connection onto granule cells, and a negative feedback loop, via a disynaptic connection in which mossy cells excite basket cells that subsequently inhibit granule cells. By altering the balance between the positive and negative feedback loops, the amount of theta and gamma in the network can be altered. Increasing the strength of the positive feedback loop relative to the mossy cell-controlled negative feedback loop increases the amount of theta. This can be achieved either by increasing the strength of the mossy cell drive to the granule cells or by decreasing the strength of the mossy cell drive to the basket cells. Affecting these synaptic weights in the opposite direction decreased theta. Thus, these simulations suggest that mossy cells within a granule cell-basket cell network can potentially serve a role in regulating theta. This potential link between theta oscillations and mossy cells has not been fully explored previously, though a former study has shown that mossy cells preferentially fire during cycles of theta (Soltesz et al., 1993). Other studies have identified phasic input from the medial septal nucleus and the nucleus of the diagonal band as sources of theta to the dentate (Stewart and Fox, 1990; Pernía-Andrade and Jonas, 2014), but as these two areas were not included in the model, they could not be the source of theta in these results.

Finally, we note that the dentate system can enter an aberrant bursting state reminiscent of epileptic activity within a relatively large range of parameter values. This is notable because much of the experimental work published on hippocampal mossy cells has been grounded in studying their role in the formation of epilepsy after blunt head trauma. Two alternate theories have been proposed. One, known as the “dormant basket cell” theory, proposes that, due to mossy cell loss that results from blunt head trauma, inhibitory interneurons that are normally innervated by mossy cells lose that excitatory input, resulting in lower levels of granule cell inhibition and thus, greater activity (Sloviter, 1991; Sloviter et al., 2003). An alternate theory, known as the “irritable mossy cell” theory, holds that not all mossy cells are lost after blunt head trauma, and that those remaining become hyper-excited, leading to over stimulation of the granule cell population (Santhakumar et al., 2000). Based on the results of the associational pathway simulations, it seems likely that both of these theories have validity, as epileptic-like activity appears both when the strength of mossy cell input to granule cells is increased and when either mossy cell input to basket cells or basket cell input to granule cells is decreased.

### Interpretation of “Clusters” of Granule Cell Activity: A Measure of Network Dynamics

Our goal is to understand how multiple, specific neurobiological mechanisms that are known to comprise the hippocampal formation interact to produce network dynamics. In this model we have incorporated as many important details as possible regarding the morphology of the dendritic and axonal processes, the biophysical properties of cellular membranes with respect to passive electrical characteristics (membrane resistance and capacitance) and active membrane processes (voltage-dependent and ion-dependent conductances), the spatial distribution of synaptic inputs in dendritic regions, the topographic distribution of synaptic connections between populations of neurons, the ratio of principal neurons to interneurons, etc. Predicting how network dynamics emerge from the interaction of these various mechanisms requires driving these network elements with an input signal. It is obvious that the nature of the interaction between components of the network – and thus, the expression of the very network dynamics in question – will depend to a large extent on the input signal itself. This problem of how to separate network dynamics from the nature of the test signal used to obtain their expression is a problem of long standing in the field of biomedical engineering (Marmarelis and Marmarelis, 1978; Sclabassi et al., 1988; Marmarelis, 2004; Marmarelis and Berger, 2005). One of the key solutions is the use of randomized inputs which provides a basis for input strength, and/or input pattern, or some other parameter of the input signal, to be equalized as much as possible. Thus, in the present analysis, the input signal was a series of impulses having randomly varying inter-impulse intervals. As a result, the input signal varies widely (in this case from 0.1 to 3,194.9 ms), and remains reasonably unbiased. The wide range of randomized input signals maximizes the probability that most mechanisms of a neural system will be activated. For example, random input signals ensure not only that monosynaptically stimulated cells are subject to frequency-dependent release of neurotransmitter (e.g., well-known paired pulse potentiation and similar phenomena) and to frequencies sufficient for activating receptor-channels with voltage-dependence or voltage-dependent blockade (e.g., NMDA), but also so that different components of a given network are activated transsynaptically through as many different pathways and circuits as possible (see Yeckel and Berger, 1998). For these reasons, the net functional consequence of using random input signals is that any resulting network behavior will reflect contributions from a great many of the mechanisms, neurons, and/or circuits comprising the network. This is why, in fact, the “clusters” of granule cell activity represent a reasonable index of network dynamics, perhaps better than any alternative index.

In addition, although being mostly analyses on the system and signal level, our model also provides a valuable platform for studying the computational and cognitive functions of the hippocampus. For example, the dentate gyrus has long been postulated to play a role in pattern separation or sparsification for memory formations (McNaughton and Morris, 1987; Treves and Rolls, 1992; O’Reilly and McClelland, 1994). However, most previous theoretical and computational studies have utilized simple connectivity schemes between the EC and dentate gyrus, and highly abstracted neuron models. The exact nature of pattern separation remains unclear. By contrast, our model is built with anatomically constrained EC-dentate connectivity and eletrophysiologically accurate neuron models, and thus can be used to quantitatively investigate how pattern separation is performed by the EC-dentate system and provide predictions that can be more readily verified with experimental studies.

## Conflict of Interest Statement

The authors declare that the research was conducted in the absence of any commercial or financial relationships that could be construed as a potential conflict of interest.
